# Role of Vitamin K in Selected Malignant Neoplasms in Women

**DOI:** 10.3390/nu14163401

**Published:** 2022-08-18

**Authors:** Anna Markowska, Michał Antoszczak, Janina Markowska, Adam Huczyński

**Affiliations:** 1 Department of Perinatology and Women’s Health, Poznań University of Medical Sciences, 60-535 Poznań, Poland; 2 Department of Medical Chemistry, Faculty of Chemistry, Adam Mickiewicz University, 61-614 Poznań, Poland; 3 Department of Oncology, Gynecological Oncology, Poznań University of Medical Sciences, 60-569 Poznań, Poland

**Keywords:** vitamin K, breast cancer, cervical cancer, ovarian cancer

## Abstract

The main function of vitamin K in the human organism is its activity in the blood clotting cascade. Epidemiological studies suggest that reduced intake of vitamin K may contribute to an increased risk of geriatric diseases such as atherosclerosis, dementia, osteoporosis, and osteoarthritis. A growing number of studies also indicate that vitamin K may be involved not only in preventing the development of certain cancers but it may also support classical cancer chemotherapy. This review article summarizes the results of studies on the anticancer effects of vitamin K on selected female malignancies, i.e., breast, cervical, and ovarian cancer, published over the past 20 years. The promising effects of vitamin K on cancer cells observed so far indicate its great potential, but also the need for expansion of our knowledge in this area by conducting extensive research, including clinical trials.

## 1. Introduction

**Figure 1:**
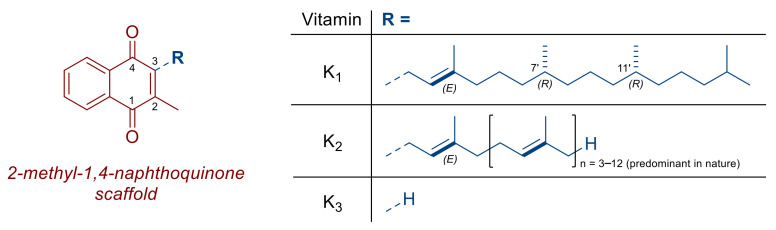
Structure of vitamin K.

Vitamin K is a group of fat-soluble organic chemical compounds containing a 2-methyl-1,4-naphthoquinone ring substituted at C-3 position by various substituents (Figure 1). Natural vitamin K occurs in two forms: (i) vitamin K_1,_ produced by green plants (phylloquinone), which has a phytyl side chain composed of three saturated and one unsaturated isoprene units at C-3 position; (ii) vitamin K_2_, a series of compounds (menaquinones) whose side chain at C-3 position is composed of multiple (mainly 4 to 13) unsaturated isoprene units (Figure 1) and produced generally by bacteria, with the exception of menaquinone-4 which is produced via biosynthetic conversion of vitamin K_1_ in the body. Menaquinones can be designated by the general formula MK-X, where X indicates the number of isoprene units in the side chain, for example, menaquinone-6 (MK-6). Vitamin K_3_ (menadione) (Figure 1) is of synthetic origin. High levels of vitamin K_1_ can be found in green leafy vegetables and some vegetable oils, while other types of vegetables, fruits, cereal grains, or their milled products are poor sources of this nutrient [1]. Animal products (meat, fish, milk products, and eggs) contain rather low levels of vitamin K_1_, but the content of menaquinones is higher in the liver. Various menaquinones are present, e.g., in fermented foods [2], shellfish, beef, pork, chicken, eggs (yolk), and butter [3], while cheese contains significant quantities of MK-8 and MK-9 [4].

Vitamin K is active in prothrombin synthesis (the process of blood clotting) and bone metabolism, by regulating calcium metabolism and reducing inflammation, but it may also have an impact on the course of malignancies [5,6,7]. However, the results of studies on the effects of vitamin K on malignancies are relatively few and inconclusive [8,9,10]. They seem to be strongly dependent on the type of cancer, the form of vitamin K used, or the type of study conducted, among other factors.

Palmer et al. [11], in a Danish prospective cohort study involving more than 56,000 men with a mean age of 56 years, examined the association between dietary vitamin K_1_ intake and cancer mortality. After adjusting for demographic and lifestyle factors, they have found that vitamin K_1_ contributes to a lower rate of cancer-caused death (Q5 vs. Q1, HR 0.80, 95% CI 0.75, 0.86) [11]. In contrast, an epidemiological study spanning more than eight years and involving a total of 361 pancreatic cancer cases showed differential effects of the supply of different forms of vitamin K on the risk of cancer development [12]. The intake of vitamin K_1_ and dihydrovitamin K_1_ appeared to reduce the risk of pancreatic cancer (Q4 vs. Q1, HR 0.57, 95% CI 0.39, 0.83), but a similarly favorable relationship was not observed for vitamin K_2_ intake [12]. Completely different results have been provided by Nimptsch et al. [13], who have indicated that dietary intake of vitamin K_2_, as opposed to vitamin K_1_, may be associated with a reduced risk of cancer occurrence and death, particularly in men.

A long-term questionnaire-based study was conducted in the US to evaluate the effects of the intake of vitamin K_1_, vitamin K_2,_ and total vitamin K on the prostate cancer risk [14]. The study included more than 2900 cases, 490 of which were at an advanced stage, but no indication has been found that vitamin K intake had a beneficial effect on reducing the incidence of this type of cancer [14]. Nevertheless, interesting therapeutic effects can be expected when vitamin K is administered in combination with commonly used cancer drugs. For example, Haruna et al. [15], who conducted a randomized phase II trial on 68 patients with hepatocellular carcinoma using sorafenib (a kinase inhibitor), noted that a prolonged median overall survival was achieved in the group of patients responding to treatment (partially or completely) and receiving vitamin K.

### Anticancer Mechanism of Action

Despite a growing body of research results, a detailed mechanism of vitamin K’s action on cancer cells remains unclear. The observed anticancer effects of vitamin K include: (i) inhibition of proliferation, (ii) induction of differentiation, (iii) inhibition of the potential for metastasis, and (iv) induction of autophagy or apoptosis. The phenomenon of vitamin K-mediated apoptosis proceeds much more slowly than that caused by conventional cancer drugs, making it possible to analyze in detail the various stages involved [16]. The mechanisms of vitamin K action on cancer cells have already been reviewed in literature [17], therefore, only selected molecular targets on which vitamin K acts in vitro and/or in vivo are presented below.

In in vitro tests, Dasari et al. [18], have evaluated the effects of vitamin K_2_ on a VCaP cell line derived from hormone-refractory prostate cancer. They observed that vitamin K_2_ significantly inhibited tumor cell proliferation in a dose-dependent manner, induced apoptosis, caspase 3/7 activity, increased levels of reactive oxygen species (ROS), and decreased androgen receptor expression [18]. A slightly different vitamin K action mechanism was described on the basis of in vitro studies on colon cancer cell lines SW480 and SW620 [19]. Vitamin K_3_ inhibited the epithelial-mesenchymal transition (EMT) and Wnt signaling pathway by affecting various molecular targets, such as cadherins, cyclins, and β-catenin [19]. Some other studies have shown that vitamin K leads to depolarization of the mitochondrial membrane and a release of cytochrome c into the cytosol with the generation of apoptosome, which drives the activation of caspase 9, ultimately leading to the activation of caspase 3 and the initiation of apoptosis [20,21,22]. In addition, vitamin K_2_ can reduce cyclin D1 expression in cancer cells by inhibiting the binding of the nuclear factor κB (NF-κB) to the cyclin D1 promoter, which occurs by arresting the cell cycle in the G1 phase [23]. Furthermore, vitamin K_2_ derivatives showed growth inhibitory effects not only on cancer cells derived from various organs but also on those resistant to radiotherapy by generating ROS [24].

As indicated in in vivo studies, vitamin K_2_ had an inhibitory effect on bladder cancer development by inducing metabolic stress; vitamin K_2_ promoted PI3K/AKT/HIF-1α pathway-dependent glycolysis, leading to AMPK-dependent autophagic cancer cell death [25]. Conversely, vitamin K_2_ inhibited hepatocellular carcinoma cell proliferation in in vivo tests, through direct binding to 17β-hydroxysteroid dehydrogenase 4 (HSD17B4), a protein that promotes cell proliferation in this cancer [26]. This resulted in inhibition of the activation of Akt and MEK/ERK signaling pathways, leading to decreased STAT3 activation [26].

The positive effects of vitamin K have been confirmed in many types of cancer. However, to the best of our knowledge, literature provides as yet no article describing the potential effects of vitamin K on the development and course of selected female malignancies. In this review paper, which is part of a series outlining the anticancer effects of vitamins on selected female malignancies [27,28,29], we focused on the potentially beneficial role of vitamin K on breast, cervical, and ovarian cancers. To this end, we have searched the Google Scholar and PubMed databases in detail for original papers describing the potential anticancer activity of vitamin K in in vitro and in vivo tests (Table 1), as well as in human observational studies published over the last two decades.

**Table 1:** In vitro (and animal) studies with vitamin K on cancer cell lines.

Cancer Type	Active Form of Vitamin K	Cancer Cell Lines Sensitive to the Action of Vitamin K	Optimal Concentration	Combination Treatment/In Vivo Studies	Reference
Breast cancer	K_2_	Hs578T, SUM159PT	5 μg mL^−1^ (supplemented medium)		[30]
	K_2_ (MK-4)	BT-474, MDA-MB-231, MDA-MB-468	10–25 μM (supplemented medium)		[31]
	K_2_ (MK-4)	MDA-MB-231	124.4 µM (IC_50_)	low-glucose medium (5.5 mM)	[32]
	K_3_	BT-474, MCF-7, MDA-MB-231, SK-BR3	11.3–25.1 µM (IC_50_)	in vivo studies	[33]
	K_3_	MCF-7	14.2 μM (IC_50_)		[34]
Cervical cancer	K_3_	SiHa (HPV-16 positive)	10.8 µM (IC_50_) 21.7 µM (IC_90_)		[35]
	K_3_	HeLa, SiHa		ultraviolet radiation A + in vivo studies	[36]
Ovarian cancer	K_2_ (MK-4)	PA-1, TYK-nu	5.0–73.0 µM (IC_50_)		[37]
	K_2_ (MK-4)	TYK-nu	73.0 µM (IC_50_)		[38]
	K_3_	OVCAR-3, SK-OV-3	7.5 µM (~59% cell death)		[39]
	K_3_ (menadione bisulfite)	MDAH 2774, CAOV-3, ES-2	22.0–41.8 µM (CD_50_)	vitamin C	[40]
	K_3_ (menadione bisulfite)	MDAH 2774	20.3 µM (supplemented medium)	vitamin C	[41]
	K_3_	SK-OV-3	20.0 µM (80% inhibition rate)		[42]

## 2. Breast Cancer

Breast cancer (BC) is the most common malignancy occurring in women. According to epidemiological data, 2,261,419 women developed BC worldwide in 2020, accounting for 24.5% of all malignancies occurring among women [43,44]. The search for new treatments to improve patients’ lives with this type of cancer is extremely important. Unfortunately, the results of studies on the effects of vitamin K on the development and course of BC are inconclusive. The inconclusiveness mainly refers to the in vitro studies concerning the evaluation of vitamin K supply in the population.

### 2.1. In Vitro and In Vivo Studies

A study by Beaudin et al. [30], showed differential effects of vitamin K on cancer cells from the triple-negative BC (TNBC) cell line. Vitamin K_1_ was observed to increase the growth of cancer cells and the expression of γ-carboxyglutamate (GLA), the matrix amino acid responsible for binding calcium cations in the cell [30]. In contrast, the exposure of Hs578T, MDA-MB-231 and SUM159PT cells to vitamin K_2_ had an antiproliferative effect and caused a decrease in cancer cell activity; however, GLA protein expression was not affected [30]. Miyazawa et al. [31], have conducted in vitro studies on established TNBC cell lines (MDA-MB-231 and MDA-MB-468) using vitamin K_2_. In doing so, they have confirmed previous findings [30], indicating that vitamin K_2_ has cytotoxic properties against this subtype of BC [31]. On the other hand, adding an inhibitor of the autophagy process, 3-methyladenine, to the cell culture has attenuated this effect [31]. This indicated the involvement of the autophagy-dependent cell death rather than typical apoptosis, which was further supported by the absence of known features of the latter process, such as chromatin condensation and caspase 3 [31]. Another study on the effects of vitamin K_2_ on TNBC has reported a significant dose-dependent effect of menaquinone-4, MK-4, that inhibited the growth of MDA-MB-231 and MDA-MB-453 cells, the HER-2^+^ BC cell line; vitamin K_2_ at concentrations ranging from 100 μM to 150 μM caused inhibition of cell growth in both the adhesion and proliferation phases [32].

Yamada et al. [33], documented the effects of vitamin K on a subcutaneous model of TNBC in a mouse study. The addition of vitamin K_3_ significantly inhibited tumor growth, in a manner dependent on the vitamin dose and exposure time, with extracellular signal-regulated kinase (ERK) playing a key role in inhibiting tumor growth induced by vitamin K_3_ application [33]. According to the authors of this publication, the observed anticancer effect should be a step forward in the development of molecular therapeutics against TNBC. Vitamin K_3_ showed cytotoxicity that induced DNA fragmentation in MCF-7 cells with IC_50_ value of 14.2 µM; detailed mechanistic studies revealed that vitamin K_3_ caused mitochondrial dysfunction, including the loss of mitochondrial membrane potential, while mitochondrial damage was induced by ROS generation and subsequent caspase 7/9 activation [34].

**Figure 2:**
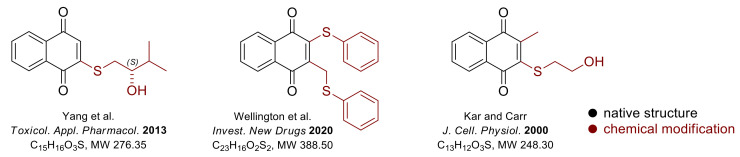
Structure of vitamin K_3_ derivatives showing promising activity against BC cell lines [45,46,47].

A variety of vitamin K_3_ derivatives also showed activity against BC cell lines [45,46], including those resistant to doxorubicin (Figure 2) [47]. For example, CR108, a vitamin K_3_ derivative, induced apoptosis via ROS and the mitochondrial damage pathway associated with p38 MAP kinase and survival, both in MCF-7 BC cells lacking HER-2 overexpression and in BT-474 cells with HER-2 overexpression [45]. Wellington et al. [46], described that other thioether derivatives of vitamin K_3_ might also show anticancer properties. The in vitro tests, using the MCF-7 BC cell line, revealed the ROS generation and disruption of the mitochondrial membrane potential, indicating that the cells underwent apoptosis [46]. The selectivity of sulfide derivatives of vitamin K against cancer cells was generally higher than against normal cells (WI-38) [46], which should encourage further research into chemical modification of vitamin K structure.

### 2.2. Effects in Cancer Patients

Wang et al. [10], have examined the effect of vitamin K intake on morbidity and mortality among women in the US population diagnosed with BC in a cohort of 2286 cases and 207 deaths from BC. Total vitamin K_2_ intake was associated with increased BC risk (Q5 vs. Q1, HR 1.26, 95% CI 1.05–1.52, *p* = 0.01) and death in a statistically significant manner (Q5 vs. Q1, HR 1.71, 95% CI 0.97–3.01, *p* = 0.04) [10]. In contrast, this relationship was not observed for vitamin K_1_ and total vitamin K intake [10]. This suggests that reduction of the amount of vitamin K_2_ in the diet could lower the risk of developing BC. Further results on the relationship between dietary vitamin K content and BC incidence have been provided by Nasab et al. [48]. They compared the indicators of dietary content in BC patients and healthy subjects; this included dietary composition with regard to both mineral (Ca, Mg, and Zn) and vitamin (A, B_2_, B_6_, B_7_, B_12_, C, E, and K) content in a group of 180 patients diagnosed with BC and 360 healthy women [48]. The authors found a significant association between the reduced risk of BC spread and the dietary vitamin K intake (OR 0.58, 0.37–0.90) [48].

## 3. Cervical Cancer

Cervical cancer (CC) is the most common malignancy in women in the countries with lower living standards. It results from a long process of changes in the normal cervical epithelium under the influence of persistent HPV infection [49,50]. As its high incidence, the search for new and effective ways to combat CC is a highly timely challenge for researchers. However, only a few papers describing the effect of vitamin K on this type of cancer can be found in the scientific literature.

### 3.1. In Vitro and In Vivo Studies

The effects of vitamin K_3_ on the morphology and volume of cancer cells, cell membrane integrity, mitochondrial membrane potential, and oxidative balance have been investigated in HPV 16-transformed CC (SiHa) cell cultures [35]. Vitamin K_3_ has been shown to induce an increase in ROS levels in SiHa cells and their morphological and biochemical changes [35]. In addition, vitamin K_3_ triggered mechanisms inducing cancer cell death by apoptosis [35]. On the other hand, Xin et al. [36], in a heterotransplant model from HeLa cells, have demonstrated the efficacy of vitamin K_3_ against CC also in in vivo tests. The study used ultraviolet A (UVA) photodynamic therapy with vitamin K_3_ as a photosensitizer [36]. The combined therapy resulted in a significant reduction in cancer cell viability in a dose-dependent manner, activation of the apoptosis pathway, and inhibition of tumor growth [36]. These observations were accompanied by an increase in cleaved caspase 3 and cleaved caspase 9 expression, as well as a decrease in the expression of the anti-apoptotic protein Bcl-2 [36].

### 3.2. Effects in Cancer Patients

A cohort study conducted in China (Sanxi CIN Cohort Study) based on dietary questionnaires on a group of 218 randomly selected subjects was aimed at the assessment of the effect of dietary vitamins on the development of cervical intraepithelial neoplasia [51]. Vitamin K was found to show a protective effect (Q2 vs. Q4, OR 1.60, 95% CI 1.05–2.44) if the dose was optimal [51]. Similarly, beneficial effects were observed of vitamin B_3_, B_6_, B_9_ and C intake, indicating that deficiencies in these nutrients may affect the development of CC.

## 4. Ovarian Cancer

Ovarian cancer (OC) has the worst prognosis of all gynecological cancers. It is estimated that about 70% of OC cases are diagnosed only in advanced clinical stages; for this reason, 5-year survival is recorded among 27% of patients in clinical stage III and only among about 13% in stage IV disease [26,52,53], justifying the need to search for new ways to combat this type of cancer.

### In Vitro and In Vivo Studies

Kim et al. [39], conducted tests on two OC cell lines (OVCAR-3, SK-OV-3) and found that vitamin K_3_ could induce apoptotic death of cancer cells through activation of the mitochondrial pathway and pathways dependent on caspase 8 and the proapoptotic cytoplasmic protein Bid. The observed proapoptotic effect of vitamin K_3_ may have been due to ROS generation and glutathione depletion [39]. Vitamin K_3_ also inhibited focal adhesion kinase (FAK)-dependent cell adhesion [39], indicating that this vitamin may be beneficial in the adjunctive treatment of OC. Furthermore, von Gruenigen et al. [40], conducted studies on established OC cell lines to determine the mechanistic and cytotoxic effects of vitamin K_3_ (and vitamin C) on this type of cancer. Anticancer activity was demonstrated by both vitamins [40]. Importantly, the use of vitamin K_3_ in combination with vitamin C resulted in a synergistic effect, blocking the G1 phase of the cell cycle, and ultimately to autoschizis, cell death with characteristics of both apoptosis and necrosis [40]. Similar results have been reported by other researchers [41]; human OC cells MDAH 2774 treated with a combination of vitamin K_3_ and vitamin C showed changes observed when the vitamins were used separately (for vitamin K_3_: damage to the cytoskeleton and self-cleavage; for vitamin C: damage to the plasma membrane). In addition, after 1-h exposure to the combination of the two vitamins, autoschizisis (43%), apoptosis (3%), and oncosis (1.9%) of the cancer cells tested were observed [41].

Xia et al. [42], examined the response of SK-OV-3 OC cells and cisplatin-resistant OC cells (SK-OV-3/DDP) to vitamin K_3_. The authors of the study showed a diverse response of these cell lines to vitamin K_3_; it induced apoptosis in SK-OV-3 cells by increasing ROS production, while SK-OV-3/DDP cells with high levels of p62 protein, involved in autophagy, redox signaling, and apoptosis, were less sensitive to its effects [42]. At the same time, it has been shown that downregulation of p62 protein expression can increase susceptibility to apoptosis in SK-OV-3/DDP cells [42]. It is important to note that SK-OV-3/DDP OC cells show higher basal Nrf2 levels than those of the parental SK-OV-3 cell line, which allows them to tolerate higher concentrations of ROS. In this context, vitamin K_3_ was identified as an agent that activates the Nrf2 signaling pathway, a key modulator of OC chemoresistance and progression [54], protecting SK-OV-3/DDP cells from the proapoptotic action of vitamin K_3_. As vitamin K_3_ is a safe nutrient for human consumption and could be an important supplement to prevent OC progression, vitamin K_3_-induced upregulation of Nrf2 pathway may protect non-tumor ovarian cells from oxidative stress damage.

In addition to vitamin K_3_, the effect of vitamin K_2_ (menaquinone-4, MK-4) against OC cells has also been assessed [38]. Vitamin K_2_ induced apoptosis in the TYK-nu cell line, associated with the release of cytochrome c and decreased Bcl-2 protein [38]. This process was inhibited by cycloheximide and starvation at a low concentration of serum [38]. In PA-1 cells, vitamin K_2_ (IC_50_ = 5.0 ± 0.7 μM) induced apoptosis due to increased TR3/Nur77 levels and its accumulation in mitochondria and cell nuclei [37]. On the other hand, SK-OV-3 cells proved resistant to vitamin K_2_ in the concentration range tested [37].

## 5. Conclusions

**Figure 3:**
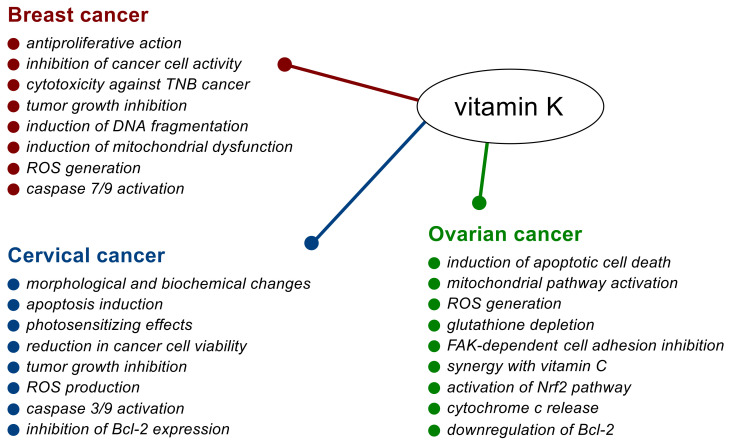
Effects of vitamin K on malignant neoplasms in women.

Vitamin K is an essential nutrient. In recent years, it has also increasingly become the subject of research into its potential use as a promising adjuvant to anticancer therapy. The effects of vitamin K on selected female malignancies, i.e., breast cancer, cervical cancer, and ovarian cancer, have so far been observed primarily in in vitro and animal tests and have indicated diverse phenotypic effects exerted by different forms of the vitamin. While most studies have focused on the effects of vitamin K_2_ and vitamin K_3_, there have been far fewer concerning vitamin K_1_. Exemplary effects of vitamin K on breast, cervical, and ovarian cancers are schematically shown in Figure 3.

Dietary vitamin intake may play an essential role in carcinogenesis. A few observational studies, mainly on breast cancer, have indicated an association between the reduced risk of morbidity, disease progression and mortality, and the dietary vitamin K intake. In addition, combined administration of vitamin K and standard chemotherapeutic drugs may contribute to better outcomes with fewer observed side effects, which could lead to improvements in efficacy and reduced costs of conventional cancer therapy. However, this approach still requires further intensive research, including clinical trials. On the other hand, in an animal study, it has been confirmed that all dietary forms of vitamin K can be converted to tissue menaquinone-4 (MK-4) [55], thus, further investigations of the physiological role of MK-4 in certain malignant neoplasms developing in women, that may be independent of classical function of vitamin K, are bound to be undertaken.

Chemical modification of the structure of vitamin K is also an exciting line of research. Various derivatives of vitamin K_3_ have proven effective against breast cancer cells, including those resistant to commonly used cytostatic drugs. This substantiates the validity of the ongoing synthetic work and justifies its continuation in the coming years.

## Data Availability

Not applicable.
